# Population-based seroprevalence of SARS-CoV-2 and the herd immunity threshold in Maranhão

**DOI:** 10.11606/s1518-8787.2020054003278

**Published:** 2020-11-27

**Authors:** Antônio Augusto Moura da Silva, Lídio Gonçalves Lima-Neto, Conceição de Maria Pedrozo e Silva de Azevedo, Léa Márcia Melo da Costa, Maylla Luanna Barbosa Martins Bragança, Allan Kardec Duailibe Barros, Bernardo Bastos Wittlin, Bruno Feres de Souza, Bruno Luciano Carneiro Alves de Oliveira, Carolina Abreu de Carvalho, Erika Barbara Abreu Fonseca Thomaz, Eudes Alves Simões-Neto, Jamesson Ferreira Leite, Lécia Maria Sousa Santos Cosme, Marcos Adriano Garcia Campos, Rejane Christine de Sousa Queiroz, Sérgio Souza Costa, Vitória Abreu de Carvalho, Vanda Maria Ferreira Simões, Maria Teresa Seabra Soares de Brito e Alves, Alcione Miranda dos Santos

**Affiliations:** I Universidade Federal do Maranhão Departamento de Saúde Pública São LuísMA Brasil Universidade Federal do Maranhão. Departamento de Saúde Pública. São Luís, MA, Brasil; II Laboratório Central do Maranhão São LuísMA Brasil Secretaria de Saúde do Estado do Maranhão. Laboratório Central do Maranhão. São Luís, MA, Brasil; III Universidade CEUMA São LuísMA Brasil Universidade CEUMA. São Luís, MA, Brasil; IV Universidade Federal do Maranhão Departamento de Medicina I São LuísMA Brasil Universidade Federal do Maranhão. Departamento de Medicina I. São Luís, MA, Brasil; V Saúde do Estado do Maranhão Hospital Presidente Vargas São LuísMA Brasil Secretaria de Saúde do Estado do Maranhão. Hospital Presidente Vargas. São Luís, MA, Brasil; VI Saúde do Estado do Maranhão São LuísMA Brasil Secretaria de Saúde do Estado do Maranhão. Assessoria. São Luís, MA, Brasil; VII Universidade Federal do Maranhão Departamento de Ciências Fisiológicas São LuísMA Brasil Universidade Federal do Maranhão. Departamento de Ciências Fisiológicas. São Luís, MA, Brasil; VIII Universidade Federal do Maranhão Departamento de Engenharia Elétrica São LuísMA Brasil Universidade Federal do Maranhão. Departamento de Engenharia Elétrica. São Luís, MA, Brasil; IX Universidade Federal do Maranhão Hospital Universitário São LuísMA Brasil Universidade Federal do Maranhão. Hospital Universitário. São Luís, MA, Brasil; X Universidade Federal do Maranhão Departamento de Engenharia da Computação São LuísMA Brasil Universidade Federal do Maranhão. Departamento de Engenharia da Computação. São Luís, MA, Brasil; XI Universidade Federal do Maranhão Curso de Medicina PinheiroMA Brasil Universidade Federal do Maranhão. Curso de Medicina. Pinheiro, MA, Brasil; XII Secretaria Municipal de Saúde São LuísMA Brasil Secretaria Municipal de Saúde. São Luís, MA, Brasil; XIII Saúde do Estado do Maranhão Centro de Informações Estratégicas de Vigilância em Saúde São LuísMA Brasil Secretaria de Saúde do Estado do Maranhão. Centro de Informações Estratégicas de Vigilância em Saúde. São Luís, MA, Brasil; XIV Universidade Federal do Maranhão Programa de Pós-Graduação em Saúde Coletiva São LuísMA Brasil Universidade Federal do Maranhão. Programa de Pós-Graduação em Saúde Coletiva. São Luís, MA, Brasil

**Keywords:** Seroepidemiologic Studies, Coronavirus Infections, Immunity, Herd, Mortality

## Abstract

**OBJECTIVE:**

To estimate the seroprevalence of SARS-CoV-2 in the state of Maranhão, Brazil.

**METHODS:**

A population-based household survey was performed, from July 27, 2020 to August 8, 2020. The estimates considered clustering, stratification and non-response. Qualitative detection of IgM and IgG antibodies was performed in a fully-automated Elecsys® Anti-SARS-CoV-2 electrochemiluminescence immunoassay on the Cobas® e601 analyzer (Roche Diagnostics).

**RESULTS:**

In total, 3,156 individuals were interviewed. Seroprevalence of total antibodies against SARS-CoV-2 was 40.4% (95%CI 35.6-45.3). Population adherence to non-pharmaceutical interventions was higher at the beginning of the pandemic than in the last month. SARS-CoV-2 infection rates were significantly lower among mask wearers and among those who maintained social and physical distancing in the last month compared to their counterparts. Among the infected, 26.0% were asymptomatic. The infection fatality rate (IFR) was 0.14%, higher for men and older adults. The IFR based on excess deaths was 0.28%. The ratio of estimated infections to reported cases was 22.2.

**CONCLUSIONS:**

To the best of our knowledge, the seroprevalence of SARS-CoV-2 estimated in this population-based survey is one of the highest reported. The local herd immunity threshold may have been reached or might be reached soon.

## INTRODUCTION

Brazil is one of the countries most severely affected by the coronavirus disease 2019 (COVID-19) pandemic. By September 21, 2020, 4,558,040 cases were reported, with 137,272 deaths ^1^_._ The national response has been controversial, testing capacity is low, and disagreements among the different levels of government over social distancing measures conveyed contradictory messages to the population. As a middle-income country, Brazil has high poverty rates and an extensive part of its population is engaged in informal activities that face difficulties to make ends meet and to follow stay-at-home measures ^2^ . As a consequence of all these facts, social distancing has not reached levels sufficient to curb and contain the COVID-19 pandemic ^3^ .

The state of Maranhão is located in the Northeast region of Brazil and has a population of 7,114,598 inhabitants in 2020 ^4^ , with an area of 329,642 km², a little larger than that of Italy. It is one of the states in Brazil, where the pandemic gathered speed early. Its first case was reported on March 20, 2020, and by September 21, 2020 the number of deaths reported was 3,664. Deaths peaked in May and decreased thereafter. From May 3, 2020 to May 17, 2020, São Luís Island, where the state capital city is located, was put into lockdown. Reduction of social mobility reached at most 55% at the end of March and during lockdown at the capital, remaining low (40%–45%) during the worst phase of the pandemic. Despite low home quarantine adherence, the number of deaths decreased, and intensive care units occupancy diminished ^5^ .

Herd immunity threshold to attain control of severe acute respiratory syndrome coronavirus 2 (SARS-CoV-2) is an ongoing debate. Although some consider it to be around 60%–70%, using the classical formula 1–1/R_0_, in which R_0_ is the basic reproductive number ^6^ , other reports have proposed that herd immunity could be as low as 10%–20% ^7^ or around 43% ^8^ , due to the heterogeneity in susceptibility or exposure to infection across population groups ^7^^,^^8^ . However, reported population-based seroprevalences of SARS-CoV-2 were lower than the herd immunity thresholds, ranging from extremely low infections rates, close to 1%–3% ^9^^,^^10^ , to values as high as 14.3% in Barcelona ^11^ , Spain, and 22.7% in New York City ^12^ . In Brazil, the highest reported population-based seroprevalences were 17.9%, for the São Paulo municipality ^13^ , and 66% for Manaus, where herd immunity may have played an important role in stablishing the size of the epidemic ^14^ .

The infection fatality rate (IFR) and the percentage of asymptomatic infections of SARS-CoV-2 are known with uncertainty. Early reports at the beginning of the pandemic estimated IFR at values between 0.6% and 1.3% ^15^^,^^16^ , and considered asymptomatic infections as being highly prevalent ^15^^,^^17^ . Most recent reviews, however, estimated a lower IFR with large variations across sites ^10^^,^^18^ and a much lower percentage of asymptomatic infections ^11^^,^^19^^,^^20^ .

Population-based surveys are necessary to monitor the infection progression, since most cases are undocumented ^21^ . However, few population-based studies on the prevalence of SARS-CoV-2 have been performed, especially in low and middle-income countries. In this population-based study, we estimated the overall seroprevalence of SARS-CoV-2 using a serum testing electrochemiluminescence immunoassay. Sociodemographic characteristics of the population, self-reported symptoms, adherence to non-pharmaceutical interventions (NPI), use of health services, previous molecular and antibody testing among the infected, and the IFR were also assessed.

## METHODS

### Study Design and Participants

A cross-sectional survey to estimate the seroprevalence of antibodies against SARS-CoV-2 was conducted from July 27, 2020 to August 8, 2020 by population-based household sampling, in cooperation between the *Universidade Federal do Maranhão* and the *Secretaria de Saúde do Estado do Maranhão,* Brazil.

Conglomerate sampling in three stratified stages in four regions was used. The regions were the Island of São Luís, including the state capital, small municipalities (< 20,000 inhabitants), medium-sized municipalities (20,000 to 100,000 inhabitants) and large municipalities except for the island (> 100,000 inhabitants). In each stratum, in the first stage, 30 census tracts were selected by systematic sampling. In the second stage, 34 households were selected in each census tract by systematic sampling. In the third stage, an eligible resident (residing for at least six months in the household) aged one year or more was selected by simple random sampling using a table of random numbers.

### Data Collection, Instruments, and Variables

Trained professionals from the municipal and state health departments were responsible for data collection. The starting point (identified with an × on the map) and the geographic boundaries of each census tracts were identified using a map provided by the Brazilian Institute of Geography and Statistics (IBGE). The first interview was held in the household closest to the starting point of each sector. Then, facing that domicile, the interviewer walked to the left with his/her right shoulder facing the wall/residences. Without including the visited house, the interviewer counted five residences and conducted the next interview in the fifth one. If the selected household was empty at the time or the elected person did not agree to participate in the survey, the next house to the left (neighbor) of the original one was taken as a replacement. If that house was also empty or if the elected person refused to participate the next house to the left was visited. Then the interviewer counted five domiciles and conducted the next interview in the fifth house after the original one. The team always proceeded to the left in relation to the last surveyed domicile and conducted the next interview in the fifth domicile. Non-residential buildings were excluded from the count. After completing the tour on the block, the interviewer facing the last visited domicile continued to the next adjacent block located to the left, always adopting the same strategy.

A questionnaire with closed-ended questions was applied in a face-to-face interview with the individual or his/her legal guardian. The questionnaire was composed of sociodemographic questions, adherence to NPI, self-reported symptoms, and the use of health services. The sociodemographic questions included sex, age group, self-reported skin color/race, head of the household's schooling, monthly family income in Brazilian Reals, and the number of the household residents. Head of the household's schooling was classified according to the International Standard Classification of Education (ISCED) 2011 into early childhood/primary/lower secondary education (levels 0–2), upper secondary/post-secondary non-tertiary education (levels 3–4), and tertiary education and beyond (levels 5–8) ^22^ . Skin color/race was categorized according to the IBGE and divided into white, brown, or black ^23^ .

Adherence to NPI at the beginning of the pandemic and in the last month included social distancing (yes, if the person never leaves home or seldom goes out, with a maximum of one outing every fifteen days, and no otherwise), wearing of face masks (yes, if the individual uses a mask on all exits and does not remove or seldom removes the mask from the face, and no otherwise), hand hygiene (yes, if the person sanitizes the hands more than six times per turn with soap or an alcohol gel, and no otherwise), and physical distancing (yes, if the individual never or hardly ever comes within 1.5 m of other people, and no otherwise).

A self-reported symptom rating, adapted from Pollán et al. (2020) ^11^ was used and the persons were classified into asymptomatic; oligosymptomatic: the presence of one to two symptoms without anosmia/hyposmia or ageusia/dysgeusia; and symptomatic: anosmia/hyposmia or ageusia/dysgeusia or more than two symptoms including fever, chills, sore throat, cough, dyspnea, diarrhea, nausea/vomiting, headache, fatigue, and myalgia.

Questions on the use of health services included if the individual looked for health services, received care when seeking health services, was hospitalized for over 24 hours, received a medical diagnosis of suspected COVID-19, performed RT-PCR for SARS-CoV-2, and performed an antibody test– point-of-care/serology for SARS-CoV-2.

Data were abstracted into the *Epicollect5 Data Collection* mobile application.

### SARS-CoV-2 Antibodies Detection

For the qualitative determination of antibodies against SARS-CoV-2, 5.0 ml of whole blood was collected, and after centrifugation at 1800 g for 15 min, the serum was obtained. Then, a highly sensitive and specific sandwich electrochemiluminescence immunoassay (Elecsys^®^ Anti-SARS-CoV-2 assay, Roche Diagnostics) was used to detect IgM and IgG antibodies against the SARS-CoV-2 nucleocapsid antigen according to the manufacturer's instruction using a fully automated Cobas^®^ e601 immunoassay analyzer (Roche Diagnostics) ^24^ .

### Sample Size Calculation

The formula used to determine the sample size in each stratum was given by

$$n=\frac{N}{N-1}\times P\times Q\times \frac{1}{CV^{2}\times P^{2}+\frac{P\times Q}{N-1}}$$

N being the population size in each stratum; P the expected prevalence in the stratum; Q=1-P; and CV the coefficient of variation of the prevalence estimates within the stratum. In each stratum, the expected prevalence of infection was 20%, and the coefficient of variation was 10%. For the final estimate, a design effect of 2 was added. Thus, the minimum number of individuals per stratum was 800, totaling 3,200 individuals to compose the sample. Predicting losses, the sample size was increased by 25% resulting in 4,000 observations.

### Statistical Analysis

The basic sample weight of each selected unit (census sector, household, and individual) was estimated separately for each stratum, considering the inverse of the selection probability according to the sampling plan specified for the study.

The probability of selection of the census sector “j” in each stratum “i” of the sample is given by 30/S_i_, in which “S_i_” is the number of census sectors of the stratum “i” in the population and the probability of the domicile of the census sector “j” of the stratum “i” being selected was obtained from the following expression: 34/D_ij_, in which “D_ij_” is the number of domiciles in sector “j” of the stratum “i” in the population. The probability of each resident in the selected household was given by 1/(number of residents in the household). The number of sectors and domiciles was obtained from the 2010 Census of the IBGE.

Since losses, refusals, and non-responses occurred, the response rate in each stratum was also estimated. Considering that there were three stages, the final weight was obtained by the product of the basic weight in each stage and the response rate.

All analyses were performed using R version 4.0.2. Weighting factors, clustering, and stratification were incorporated into the analyses via the R survey package. Prevalence and 95% confidence interval (95%CI) of SARS-CoV-2 infection was obtained according to the sociodemographic characteristics, adherence to NPI, self-reported symptoms, and the use of health services. The chi-square test, considering the study design, was used to compare the prevalence between groups. The McNemar test was used to compare adherence to NPI over time.

The overall and sex- and age-specific IFR were estimated by dividing the estimated number of deaths by the estimated proportion of infections obtained by the serological survey multiplied by the stratified age and sex population estimates ^4^ . The number of deaths that occurred up to August 8, 2020 was abstracted from official sources ^5^ . The number of deaths occurring daily was estimated using Nowcasting by Bayesian Smoothing (NobBS), to consider reporting delays. This procedure incorporates uncertainty both in the delay distribution and in the evolution of the pandemic curve over time, resulting in smooth, time-correlated estimates of the number of deaths ^25^ . Simulations were conducted using the NobBS R package, with a negative binomial model with an adaptation phase of 10,000 iterations and a burn-in of 10,000 iterations for estimating deaths in the state of Maranhão, and the same parameters with 5,000 iterations for the São Luís Island. Furthermore, since there is underascertainment of deaths due to COVID-19, IFR was also estimated considering excess mortality due to all natural causes. Excess deaths were abstracted from the Panel to analyze the excess mortality from natural causes in Brazil in 2020 ^26^ . Data on excess mortality is only available stratified by sex and two age groups (< 60 and ≥ 60 years) ^27^ . The 95% confidence intervals for the IFR were based on delta methods accounting for the binomial variance in the numerator (number of deaths) and the estimated variance, considering the complex sampling design in the denominator (number of infections) ^28^ .

### Ethical Approval

Ethical approval was obtained from the Research Ethics Committee of the Carlos Macieira Hospital of the Maranhão State Health Secretariat under CAAE number 34708620.2.0000.8907. An informed consent form was provided by the participants or the parents/legal guardians.

## RESULTS

A total of 3,289 individuals (80.6%) agreed to participate in our study. After the exclusion of samples with insufficient material or hemolyzed samples, and cases, in which it was not possible to link the result of the examination with the person, 3,156 participants had their blood samples analyzed (77.4%). Comparing the sampling with the population distribution (age and sex estimates for 2020), men and people of working age were underrepresented in the sample.

Seroprevalence of total antibodies against SARS-CoV-2 was 40.4% (95%CI 35.6-45.3) in the state of Maranhão, Brazil. Seroprevalence varied by region, from 20.0% in small municipalities with < 20,000 inhabitants, reaching 47.6% in medium-sized municipalities from 20,000 to 100,000 inhabitants (p = 0.006). Seroprevalence in the São Luís Island, including the capital city, was 38.9%. There were no significant differences in the prevalence according to the sex or age group ( Table 1 ).

**Table 1:** Prevalence of antibodies against SARS-CoV-2 by region, sex, age group, race, schooling, family income and number of residents, state of Maranhão, Brazil, 2020

Variables		n	% weighted	Population distribution (%)	f infected	% infected weighted (95%CI)	p
Total		3156	100.0	100.0	1167	40.4 (35.6−45.3)	
Region							0.006
	São Luís Island including the capital	737	25.5	20.2	349	38.9 (24.5−53.2)	
	Municipalities with < 20,000 inhabitants	754	20.0	21.4	215	31.0 (24.3−37.8)	
	Municipalities with 20,000 to 100,000 inhabitants	839	41.6	45.1	346	47.6 (42.0−53.1)	
	Municipalities with > 100,000 inhabitants	826	12.9	13.2	257	35.2 (26.1−44.3)	
Sex							0.134
	Male	1200	37.1	49.1	426	37.2 (31.8−42.6)	
	Female	1956	62.9	50.9	741	42.4 (36.1−48.6)	
Age group (years) a							0.230
	1−9	124	5.2	16.5	49	42.6 (33.8−51.3)	
	10−19	330	14.7	18.6	125	43.0 (33.5−52.4)	
	20−29	427	12.5	18.0	184	49.2 (41.1−57.3)	
	30−39	475	15.0	16.0	170	44.4 (37.4−51.4)	
	40−49	502	16.3	12.0	191	32.2 (23.4−41.0)	
	50−59	501	14.6	8.5	168	39.1 (32.1−46.1)	
	60−69	409	11.7	5.7	144	40.3 (29.1−51.4)	
	≥ 70	386	9.8	4.8	136	34.3 (25.7−42.9)	
Self-reported skin color/race b							0.080
	White	590	20.0	-	200	32.2 (20.1−44.4)	
	Brown	2100	67.4	-	767	41.3 (37.1−45.4)	
	Black	396	12.6	-	177	49.1 (42.3−55.9)	
Head of the household's schooling (years)*							0.011
	Primary/Lower secondary	1369	37.7	-	487	40.9 (35.4−46.4)	
	Upper secondary	1251	41.9	-	512	46.2 (41.3−51.2)	
	Tertiary	517	20.4	-	161	27.5 (16.9−38.1)	
Monthly family income (Brazilian Real) a , c							0.101
	< 1000	607	17.7	-	222	40.8 (34.6−46.9)	
	1000 a < 2000	1405	42.5	-	540	45.9 (41.0−50.9)	
	2000 a < 3000	617	20.8	-	243	42.9 (35.7−50.2)	
	> 3000	493	19.0	-	155	27.9 (17.0−38.9)	
Number of residents							0.028
	1	386	3.9	-	122	35.4 (27.2−43.6)	
	2	840	15.7	-	302	37.7 (32.0−43.5)	
	3	739	21.2	-	297	44.9 (40.0−49.8)	
	4	628	28.0	-	234	38.8 (29.0−48.5)	
	≥ 5	563	31.2	-	212	40.9 (33.5−48.3)	

95%CI: 95% confidence interval; SARS-CoV-2: severe acute respiratory syndrome coronavirus 2.

a Numbers did not add up to total because of missing values.

b Yellow and indigenous were excluded because they were too few for a meaningful analysis.

c 1 Brazilian Real (R$) is equivalent to approximately US$ 5.60 US dollars.

White people had a lower point prevalence (20.0%) when compared with both brown (41.3%) and black people (49.2%), but of borderline significance (between 0.05 and 0.10). Persons with tertiary education had a lower prevalence of infection (27.5%) than their counterparts (p = 0.011). Although point prevalence was lower among those with a monthly family income above 3,000 Brazilian Reals, the difference did not reach a significant level. Infection rates were higher among households with three dwellers (44.9%) (p = 0.028) ( Table 1 ).

Population adherence to NPI to contain the COVID-19 pandemic were mostly higher at the beginning of the pandemic than in the last month. Social distancing decreased from 52.7% to 37.4% (p < 0.001). The percentage of wearing a face mask decreased from 61.4% to 55.5% (p < 0.001). Differences in infection rates between those that maintained social distancing and those that did not were evident both at the beginning of the pandemic (36.4% vs 45.0%, p = 0.020) and in the last month (34.0% vs 44.3%, P = 0.015). SARS-CoV-2 infection rates were significantly lower in the last month among mask wearers and those that maintained a distance of at least 1.5 m from other people compared to their counterparts (p = 0.036 for mask-wearing and p = 0.030 for physical distancing) ( Table 2 ).

**Table 2:** Prevalence of antibodies against SARS-CoV-2 according to adherence to non-pharmaceutical interventions at the beginning of the pandemic and in the last month, state of Maranhão, Brazil, 2020

Non-pharmaceutical interventions			n	% weighted	f infected	% infected weighted (95%CI)	p
At the beginning of the pandemic							
	Social distancing						0.020
		No	1392	47.3	557	45.0 (39.3−50.6)	
		Yes a	1764	52.7	610	36.4 (30.6−42.2)	
	Wearing of face masks						0.395
		No	1153	38.6	423	42.3 (35.8−48.8)	
		Yes b	2003	61.4	744	39.3 (33.7−44.8)	
	Hand hygiene						0.285
		No	1455	47.9	554	42.7 (36.9−48.4)	
		Yes c	1701	52.1	613	38.4 (31.9−44.9)	
	Physical distancing						0.065
		No	1548	52.2	602	43.5 (37.6−49.4)	
		Yes d	1608	47.8	565	37.1 (31.4−42.8)	
Last month							
	Social distancing						0.015
		No	1875	62.6	757	44.3 (39.6−49.0)	
		Yes a	1281	37.4	410	34.0 (26.5−41.4)	
	Wearing of face masks						0.036
		No	1310	44.5	517	45.9 (40.6−51.3)	
		Yes b	1846	55.5	650	36.0 (29.1−43.0)	
	Hand hygiene						0.095
		No	1557	51.6	612	44.4 (39.1−49.7)	
		Yes c	1599	48.4	555	36.2 (28.7−43.8)	
	Physical distancing						0.030
		No	1817	61.0	710	43.3 (38.0−48.6)	
		Yes d	1339	39.0	457	35.9 (29.7−42.2)	

95%CI: 95% confidence interval; SARS-CoV-2: severe acute respiratory syndrome coronavirus 2.

a Never leaves home or seldom goes out, with a maximum of one outing every fifteen days.

b Uses mask on all exits and does not remove or seldom removes the mask from the face.

c Sanitizes the hands ≥ 6 times per turn (morning, afternoon, and night) with soap or alcohol gel.

d Never or hardly ever comes within 1.5 m of other people.

Differences in the self-reporting symptoms were highly significant comparing those with and without antibodies to SARS-CoV-2. Among the infected, 62.2% had more than three symptoms, whereas 26.0% were asymptomatic and, 11.8% reported only one or two symptoms (oligosymptomatic). The predominant symptoms among those who tested positive for SARS-CoV-2 were anosmia/hyposmia (49.5%), ageusia/dysgeusia (47.7%), fever (45.6%), headache (45.4%), myalgia (43.6%), and fatigue (41.1%) ( Table 3 ).

**Table 3:** Reported symptoms of SARS-CoV-2 infection, state of Maranhão, Brazil, 2020

Variables		Non-infected (n = 1,989)		Infected (n = 1,167)		p
		n	% weighted (95%CI)	n	% weighted (95%CI)	
Self-reported symptom rating a						< 0.001
	Asymptomatic	1104	52.1 (47.3−56.9)	320	26.0 (21.0−31.0)	
	Oligosymptomatic (1 to 2 symptoms)	427	22.8 (18.8−26.7)	134	11.8 (8.9−14.6)	
	Symptomatic (≥ 3 symptoms)	458	25.2 (21.8−28.5)	713	62.2 (56.2−68.3)	
Self-reported symptoms						
	Fever	296	16.7 (13.1−20.4)	494	45.6 (39.9−51.3)	< 0.001
	Shivers	253	13.7 (9.9−17.6)	379	34.3 (29.5−39.2)	< 0.001
	Sore throat	345	18.3 (14.7−22.0)	378	34.5 (30.1−39.0)	< 0.001
	Cough	356	17.6 (13.7−21.5)	369	33.1 (29.7−36.5)	< 0.001
	Dyspnoea	184	10.9 (8.4−13.4)	209	18.6 (15.0−22.2)	0.001
	Runny nose	370	19.2 (16.0−22.3)	364	32.2 (28.0−36.5)	< 0.001
	Palpitations	204	9.5 (7.1−11.9)	178	15.6 (12.1−19.2)	< 0.001
	Anosmia/Hyposmia	117	7.3 (5.0−9.6)	547	49.5 (42.5−56.5)	< 0.001
	Ageusia/Dysgeusia	133	8.2 (5.8−10.5)	535	47.7 (40.4−54.9)	< 0.001
	Diarrhoea	186	8.9 (6.5−11.2)	210	18.1 (15.1−21.2)	< 0.001
	Nausea/vomiting	146	7.2 (5.2−9.2)	177	15.1 (12.0−18.2)	< 0.001
	Headache	509	27.5 (22.7−32.2)	491	45.4 (38.7−52.0)	< 0.001
	Abdominal pain	201	10.8 (7.7−13.9)	196	19.8 (14.5−25.1)	0.009
	Myalgia	368	17.9 (14.0−21.8)	485	43.6 (36.8−50.3)	< 0.001
	Fatigue	333	17.1 (13.0−21.3)	449	41.1 (34.5−47.7)	< 0.001
	Loss of appetite	217	11.1 (8.2−14.0)	396	35.2 (29.2−41.2)	< 0.001

95%CI: 95% confidence interval; SARS-CoV-2: severe acute respiratory syndrome coronavirus 2.

a Asymptomatic: no symptoms; oligosymptomatic: presence of 1 to 2 symptoms without anosmia/hyposmia or ageusia/dysgeusia; symptomatic: anosmia/hyposmia or ageusia/dysgeusia or more than 2 symptoms including fever, chills, sore throat, cough, dyspnea, diarrhea, nausea/vomiting, headache, fatigue, and myalgia.

Among the infected, 27.6% sought medical care and most received it. A small minority (1.9%) was hospitalized for over 24 hours, 13.3% were told they were suspected of having COVID-19, 4.3% performed an RT-PCR for SARS-CoV-2, and 13.5% performed a point of care test/serology for SARS-CoV-2 ( Table 4 ).

**Table 4:** Use of health services by individuals with SARS-CoV-2 antibodies, state of Maranhão, Brazil, 2020

Variables		n	%	95%CI
Looked for health service				
	No	888	72.4	64.6−80.3
	Yes	279	27.6	19.7−35.4
Received care when sought health service				
	Yes	239	19.0	14.9−23.0
	No	40	8.6	0.0−18.1
	Did not look for health service	888	72.4	64.6−80.3
Hospitalized for over 24 hours				
	No	1148	98.1	96.9−99.2
	Yes	19	1.9	0.8−3.1
Received a medical diagnosis of suspected COVID-19				
	No	1014	86.7	83.1−90.2
	Yes	153	13.3	9.8−16.9
Performed RT-PCR for SARS-CoV-2				
	No	1123	95.7	93.5−98.0
	Yes	44	4.3	2.0−6.5
Performed antibody test (point-of-care/serology) for SARS-CoV-2				
	No	1019	86.5	82.8−90.1
	Yes	148	13.5	9.9−17.2
	Total	1167	100.0	

95%CI: 95% confidence interval; SARS-CoV-2: severe acute respiratory syndrome coronavirus 2.

The IFR was 0.14% for the state of Maranhão, and 0.28% for the São Luís Island, considering reporting delays by NobBS. IFR was higher for men and older adults ( Table 5 ). The estimate doubled to 0.28%, using data on excess mortality ( Table 6 ). The case reporting rate was 4.5% for the state of Maranhão, and 3.4% for the São Luís Island, resulting in a ratio of the estimated infection to the reported cases as 22.2 for the state of Maranhão, and 29.9 for the São Luís Island (data not shown).

**Table 5:** Estimated number of infections, deaths, and infection fatality rates of SARS-CoV-2 by sex and age groups, state of Maranhão and São Luís island, Brazil, 2020

Sex	Age Group, years	Estimated number of infections	Number of deaths (estimated by nowcasting)	Infection fatality rate, % (95%CI)
Male			
	0−9	274,241	8	0.00 (0.00−0.01)
	10−19	236,559	12	0.00 (0.00−0.01)
	20−29	303,111	21	0.01 (0.00−0.01)
	30−39	198,478	76	0.04 (0.03−0.05)
	40−49	100,908	150	0.15 (0.10−0.21)
	50−59	101,695	274	0.27 (0.18−0.40)
	60−69	64,637	584	0.90 (0.68−1.20)
	≥ 70	67,000	1381	2.06 (1.56−2.72)
	Total	1,299,992	2506	0.19 (0.17−0.22)
Female			
	0−9	228,212	14	0.01 (0.00−0.01)
	10−19	323,171	5	0.00 (0.00−0.00)
	20−29	320,049	16	0.01 (0.00−0.01)
	30−39	282,436	56	0.02 (0.01−0.03)
	40−49	155,868	81	0.05 (0.04−0.08)
	50−59	132,770	163	0.12 (0.09−0.16)
	60−69	94,033	345	0.37 (0.26−0.52)
	≥ 70	48,838	898	1.84 (1.25−2.69)
	Total	1,533,005	1579	0.10 (0.09−0.12)
Overall			
	0−9	500,448	22	0.00 (0.00−0.01)
	10−19	567,266	16	0.00 (0.00−0.00)
	20−29	628,088	37	0.01 (0.00−0.01)
	30−39	505,975	132	0.03 (0.02−0.03)
	40−49	275,270	231	0.08 (0.06−0.11)
	50−59	237,395	437	0.18 (0.15−0.22)
	60−69	162,429	930	0.57 (0.43−0.76)
	≥ 70	116,065	2279	1.96 (1.53−2.52)
	Total	2,877,454	4085	0.14 (0.13−0.16)
São Luís Island			
Overall	Total	556,611	1544	0.28 (0.19−0.40)

IFR: infection fatality rate; SARS-CoV-2: severe acute respiratory syndrome coronavirus 2.

**Table 6:** Estimated number of infections, excess deaths, and infection fatality rates of SARS-CoV-2 by sex and age groups, state of Maranhão, Brazil, 2020

Sex	Age Group, years	SARS-CoV-2 seroprevalence, % (95%CI)	Estimated number of infections	Number of deaths (estimate based on excess deaths due to natural causes) a	Infection fatality rate, % (95%CI)
Male					
	0−59	36.38 (30.54−42.22)	1,149,733	1366	0.12 (0.10−0.14)
	≥ 60	39.85 (32.74−46.95)	133,937	3903	2.91 (2.43−3.49)
	Total	37.18 (31.81−42.55)	1,299,992	5270	0.41 (0.35−0.47)
Female					
	0−59	44.05 (38.71−49.40)	1,415,266	563	0.04 (0.03−0.05)
	≥ 60	36.02 (24.46−47.58)	146,117	2278	1.56 (1.13−2.15)
	Total	42.37 (36.11−48.63)	1,533,005	2840	0.19 (0.16−0.22)
Overall					
	0−59	41.26 (36.84−45.69)	2,629,556	1929	0.07 (0.07−0.08)
	≥ 60	37.54 (29.24−45.85)	278,482	6181	2.22 (1.78−2.77)
	Total	40.44 (35.57−45.32)	2,877,454	8110	0.28 (0.25−0.32)

IFR: infection fatality rate; SARS-CoV-2: severe acute respiratory syndrome coronavirus 2.

a Fonte: Conselho Nacional de Secretários de Saúde. Painel de análise do excesso de mortalidade por causas naturais no Brasil em 2020. Brasília, DF: CONASS; 2020 [cited 2020 Sept 21]. Available from: https://www.conass.org.br/indicadores-de-obitos-por-causas-naturais/

**Figure 1:**
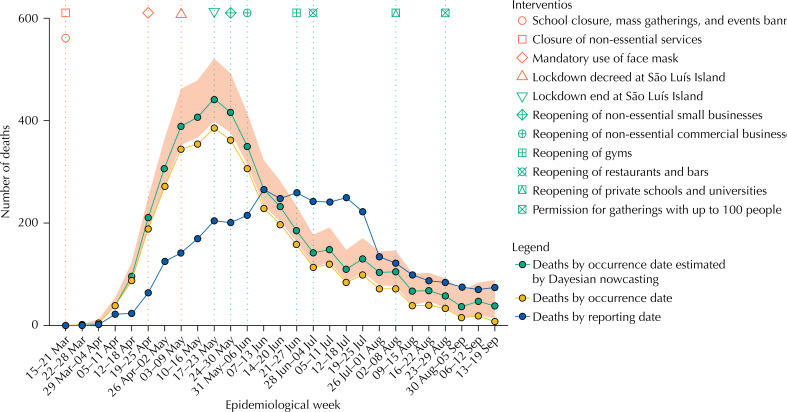
Weekly number of deaths by occurrence and reporting date, and estimated by Bayesian nowcasting from March 15 to September 19, state of Maranhão, Brazil, 2020.

**Figure 2:**
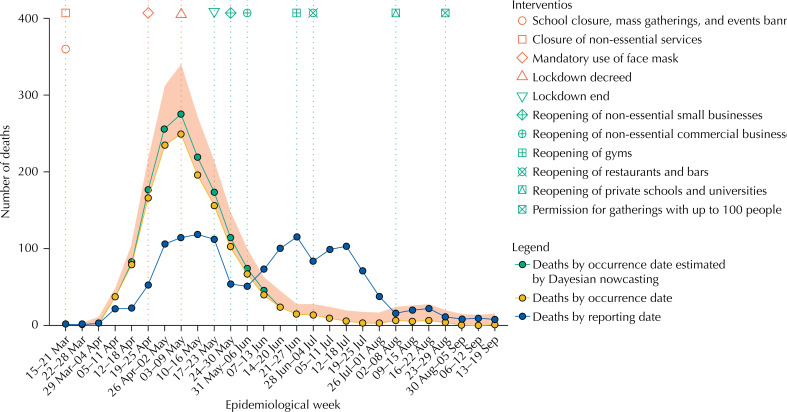
Weekly number of deaths by occurrence and reporting date, and estimated by Bayesian nowcasting from March 15 to September 19, São Luís Island, state of Maranhão, Brazil, 2020.

Figure 1 shows dates of introduction of compulsory NPI, the weekly number of deaths by dates of occurrence and reporting and estimates of the weekly number of deaths based on NobBS, considering reporting delays. The pandemic peaked from May 17, 2020 to May 23, 2020 in the state of Maranhão and from May 3, 2020 to May 9, 2020 in the São Luís Island ( Figure 2 ). Since then, the number of deaths has been decreasing, and economic activity has been gradually increasing whereas most restrictions, apart from banning mass gatherings and opening of public schools and universities, have been eased. Nearly three months since the beginning of the relaxation of social distancing, and despite increasing community mobility, reported deaths analyzed by date of occurrence remain low.

## DISCUSSION

The population-based seroprevalence of SARS-CoV-2 in the state of Maranhão, Brazil was 40.4%. We believe this is the first population-based study to report a prevalence rate in this range, for an area as big as Italy.

Over 90% of all infected people develop detectable antibodies against SARS-CoV-2 two weeks after infection ^29^ . Moreover, SARS-CoV-2 leads to robust memory T cell responses, suggesting that infection may at least prevent subsequent severe disease ^30^ . Furthermore, cross-reactivity between SARS-CoV-2 and coronaviruses that cause the common cold may elicit additional protection against infection ^31^ . Due to all these factors and based on a high seroprevalence of 40.4% achieved in the survey, our data suggests that the local herd immunity threshold may have been reached or might be reached soon, depending on the patterns of heterogeneity in susceptibility or exposure to infection ^7^^,^^8^ .

Nevertheless, the achievement of herd immunity will not be sustained if protection wanes ^32^ . Thus, durable immunity may not be attained before vaccination, and consequently, the population would remain susceptible to future recurrent outbreaks ^6^ .

In our study, we used the Elecsys® Anti-SARS-CoV-2 electrochemiluminescence immunoassay, which presented a high specificity rate of 99.7% (95%CI 99.2-100.0) and a positive predictive value (PPV) of 97.4% with a 10% seroprevalence rate ^33^ . Electrochemiluminescence immunoassays have been shown to present higher sensitivity than lateral flow immunoassays ^34^ . Some existing lateral flow immunoassays do not attain an ideal performance to be used in seroprevalence surveys, especially if they are used with finger-prick ^35^ . Therefore, since the test we used is more sensitive and specific, we could detect a higher percentage of people with antibodies against SARS-CoV-2 with few false-positive results. The distribution and percentage of self-reported symptoms among the infected in our survey were similar to what has been reported by others ^9^^,^^11^^,^^36^ , providing further evidence that a high false-positive rate in our study is unlikely. However, a negative Roche's Anti-SARS-CoV-2 serology assay does not rule out infection ^37^ . Moreover, sensitivity may decline over time due to seroreversion ^38^ . Therefore, we may have underascertained the true SARS-CoV-2 infection rate.

We could not find evidence that infection rates differ by sex, age group, skin color, or income; however, given the survey's complex sampling design, our sample size lacked the statistical power to answer these questions. The infection rates were lower among those with tertiary education, in agreement with the São Paulo study ^39^ .

Infection rates were lower among mask wearers and among those that maintained social and physical distancing, suggesting that the use of face masks ^40^ and social ^41^^,^^42^ and physical distancing ^40^ were necessary to prevent further infections and deaths. However, adherence to NPI to curb the COVID-19 pandemic tended to diminish.

Infected people were mostly symptomatic (62.2%), and anosmia/hyposmia and ageusia/dysgeusia were the two most reported symptoms. Most cases were mild. These findings are in agreement with recent studies ^19^^,^^36^ .

Our estimate of the IFR for the state of Maranhão was lower than the rate (0.71%) estimated for Brazil ^9^ , the 0.90% estimate described for the UK ^36^ and the combined estimate of 0.68% from a meta-analysis by Meyerowitz-Katz et al. (2020) ^18^ , but more in line with the 0.24% combined estimate obtained by Ioannidis (2020) ^10^ and with the range of 0.30%-0.50% estimated by Bayesian Network Analysis ^16^ . Variations in IFR may be due to differences in the testing capacity, age structures, selective testing of high-risk populations, patterns of how deaths are attributed to COVID-19 ^6^ , and strain on the health services ^43^ . Therefore, IFR is likely to vary across populations. However, the IFR in Maranhão is one of the lowest reported to date ^10^ , even after considering reporting delays and excess deaths.

In our study, the case reporting rate was 4.5% for the state of Maranhão and 3.4% for the São Luís Island, resulting in a ratio of the estimated infection to the reported cases as 22.2 for the state of Maranhão and 29.9 for the São Luís Island. These ratios were higher than the value of 10.3 reported for Brazil ^9^ .

Our study has strong points: it is population-based, had a high response rate of 77.4%, and the use of a serum electrochemiluminescence immunoassay testing instead of a lateral flow immunoassay with finger-prick. There are some limitations: for some estimates, the confidence intervals were wide, and thus our power to detect statistically significant associations was lower than that desired; some population groups (men and people of working age) were underrepresented in our sample.
